# Relationship between neutrophil-lymphocyte ratio and insulin resistance in newly diagnosed type 2 diabetes mellitus patients

**DOI:** 10.1186/s12902-015-0002-9

**Published:** 2015-03-02

**Authors:** Meiqin Lou, Peng Luo, Ru Tang, Yixian Peng, Siyuan Yu, Wanjing Huang, Lei He

**Affiliations:** Department of Endocrinology, Zhujiang Hospital, Southern Medical University, # 253 Industry Road, Guangzhou, China

**Keywords:** Neutrophil-lymphocyte ratio, Insulin resistance, Inflammation, Diabetes mellitus

## Abstract

**Background:**

Insulin resistance (IR) plays a vital role in the pathogenesis of Type 2 Diabetes Mellitus (T2DM). The mechanism of IR may be associated with inflammation, whereas the neutrophil-lymphocyte ratio (NLR) is a new indicator of subclinical inflammation. Scholars have rarely investigated the relationship between IR and NLR. This study aims to evaluate the relationship between IR and NLR, and determine whether or not NLR is a reliable marker for IR.

**Methods:**

The sample consists of a total of 413 patients with T2DM, 310 of whom have a HOMA-IR value of > 2.0. The control group consists of 130 age and BMI matched healthy subjects.

**Results:**

The NLR values of the diabetic patients were significantly higher than those of the healthy control (P < 0.001), and the NLR values of the patients with a HOMA-IR value of > 2.0 are notably greater than those of the patients with a HOMA-IR value of ≤ 2.0 (P < 0.001). Pearson correlation analysis showed a significant positive correlation of NLR with HOMA-IR (r = 0.285) (P < 0.001). Logistic regression analysis showed that the risk predictors of IR include NLR, TG and HbA1c. NLR (P < 0.001, EXP(B) = 7.231, 95% CI = 4.277–12.223) levels correlated positively with IR. The IR odds ratio increased by a factor of 7.231 (95% CI, 4.277–12.223) for every one unit increase in NLR.

**Conclusions:**

Increased NLR was significantly associated with IR, and high NLR values may be a reliable predictive marker of IR.

**Electronic supplementary material:**

The online version of this article (doi:10.1186/s12902-015-0002-9) contains supplementary material, which is available to authorized users.

## Background

Type 2 Diabetes Mellitus (T2DM) is characterized by insulin resistance and is associated with obesity and cardiovascular diseases [1]. Several studies that explored the relationship between systemic inflammation and cardiovascular diseases [2] indicated that chronic inflammation promotes the acceleration of diabetic microangiopathy in addition to the development of macroangiopathy in diabetic patients [3,4]. Insulin resistance (IR) is a reduction in reaction or sensitivity to insulin and is considered to be the common cause of impaired glucose tolerance, diabetes, obesity, dyslipidemia, and hypertensive diseases. IR syndrome is associated with multiple metabolic disorders and was renamed metabolic syndrome by Zimmet et al. in 1997 [5]. The exact molecular action leading to IR is not yet understood, but several studies have confirmed the relationship between systemic inflammation and insulin resistance, in which an altered immune system plays a decisive role in the pathogenesis of DM [6]. The immune response to various physiological challenges is characterized by increased neutrophil and decreased lymphocyte counts, and NLR is often recognized as an inflammatory marker to assess the severity of the disease [7,8].

The count of white blood cell (WBC) is a basic but cheap, readily available, and sensitive indicator of the inflammatory status [9]. WBCs are positively associated with inflammation, particularly in cardiovascular diseases [10]. An increase in the number of neutrophils is associated with thrombus formation and ischemic injury [11-13]. WBC subtypes may reflect different aspects of infection or inflammatory processes. In recent years, the presence of neutrophilia and relative lymphopenia was shown to be an independent predictor of mortality in patients with acute heart failure [13,14]. Moreover, NLR was introduced as a novel marker to determine inflammation in cardiac and noncardiac disorders [15,16].

However, there have been few studies evaluating the prognostic value of NLR in IR, especially patients who were newly diagnosed diabetes. In this study that lies in the selection of newly diagnosed diabetic subjects. We aim to evaluate the relationship between IR and inflammation by using NLR, and determine whether NLR can be used as a predictive and reliable marker.

## Methods

### Study population and design

Conducted from July 2013 to January 2014 in the Department of Endocrinology of Zhujiang Hospital, Southern Medical University and Mingjing Diabetes Hospital, Guangdong, China, our study included 413 patients newly diagnosed with T2DM but without hypertension, any acute inflammation, infection, acute or chronic renal failure, chronic liver, heart diseases, or any microvascular complications of diabetes. The control group was comprised of 130 aged-matched healthy subjects. All participants were surveyed on the followings: age, sex, hyperlipidemia, smoking, family history, chronic disease, dietary compliance, used drugs, and other risk factors. The study protocols were conducted in accordance with the tenets of the Declaration of Helsinki and approved by the Medical Ethics Committee of Zhujiang Hospital of Southern Medical University and Mingjing Diabetes Hospital. Written informed consent was obtained from all patients.

### Measurement of NLR and IR

NLR was calculated as the simple ratio between the absolute neutrophil and lymphocyte count, which were both obtained from the same automated blood sample. NLR was computed for each subject. IR was also computed for each patient. The homeostasis model of IR (HOMA-IR) was used as a measure of IR [17]. HOMA-IR was calculated using the following formula: fasting plasma glucose (mmol/L) multiplied by fasting serum insulin (mIU/L) divided by 22.5. A HOMA-IR value of > 2.0 was indicative of IR.

### Definitions

Diabetes was diagnosed based on the World Health Organization consulting criteria [18] (i.e., fasting plasma glucose [FPG] of ≥7.0 mmol/L [126 mg/dL] and/or a 2-hpost-glucose value of ≥11.1 mmol/L [200 mg/dL]).

### Statistical analysis

For continuous variables with normal distributions, data were expressed as mean ± standard deviation. Categorical variables were expressed as percentages. The Kolmogorov–Smirnov test was used to evaluate the distribution of variables. Student’s *t*-test (independent-sample *t*-test) was used for continuous variables with normal distribution, whereas the Mann–Whitney *U* test was used for continuous variables without normal distribution. The *χ*^2^ test was used for categorical variables. Pearson’s correlation analyses were used to assess the relationships. Logistic regression analysis was used to assess the associations between IR and the other parameters evaluated. A value of P < 0.05 was accepted as level of significance (two-tailed). The SPSS statistical software (SPSS for Windows, version19.0; SPSS, Inc, Chicago, IL) was used for statistical calculations.

## Results

The groups were similar in terms of age, gender, body mass index, and smoking habits (P > 0.05). All baseline clinical characteristics of the groups are listed in Table 1. The NLR values of the patients were significantly higher than those of healthy subjects. The patient group also showed significantly higher triglyceride (TG) values and HbA1c values than the control group. No significant differences in Cr, TC, HDL, and LDL levels were detected between the patient and control groups.

**Table 1:** **Demographic and laboratory data of the patient and control groups**

**Variable**	**Diabetes patients w/o IR**	**Diabetes patients w/ IR**	**Healthy subjects**	**P value**
	**(** ***n =*** **103)**	**(** ***n*** **= 310)**	**(** ***n*** **= 130)**	
Age (years)	63.55 ± 4.60	64.24 ± 4.66	64.39 ± 6.17	0.402
Gender^a^				0.924
Male	25 (24.3)	74 (23.9)	29 (22.3)	
Female	78 (75.7)	236 (76.1)	101 (77.7)	
BMI (kg/m2)	23.65 ± 3.66	24.20 ± 3.86	23.76 ± 3.53	0.333
Smoking^a^	11 (10.7)	39 (12.7)	45 (11.5)	0.857
Cr (μmol/L)	78.56 ± 20.12	78.80 ± 17.79	77.15 ± 18.32	0.571
TG (mmol/L)	1.61 ± 0.93	2.16 ± 1.53	1.72 ± 1.53	<0.001
TC (mmol/L)	5.32 ± 1.10	6.00 ± 6.31	5.57 ± 1.42	0.413
HDL (mmol/L)	1.37 ± 0.22	1.42 ± 0.33	1.44 ± 0.83	0.527
LDL (mmol/L)	2.92 ± 0.80	3.15 ± 0.90	3.09 ± 0.91	0.083
Fins (mmol/L)	4.97 ± 1.87	14.25 ± 10.72	8.21 ± 6.23	<0.001
FPG (mmol/L)	6.55 ± 2.09	8.84 ± 3.97	5.25 ± 2.26	<0.001
HbA1c (mmol/L)	7.25 ± 1.84	8.02 ± 2.29	5.99 ± 1.49	<0.001
Microalbuminuria	17.67 ± 24.98	25.58 ± 35.73	19.00 ± 23.91	0.030
IR	1.35 ± 0.41	5.32 ± 4.95	1.87 ± 1.70	<0.001
NLR	1.71 ± 0.50	2.37 ± 0.61	1.42 ± 0.30	<0.001
Neutrophil	3.53 ± 1.33	4.17 ± 3.56	2.76 ± 0.94	<0.001
Lymphocyte	2.12 ± 0.68	1.81 ± 1.15	2.21 ± 0.35	<0.001
WBC	5.72 ± 1.54	6.20 ± 1.62	5.19 ± 1.16	<0.001

All parameters were expressed as mean ± SD (minimum–maximum) values unless otherwise stated. P < 0.05 was accepted as the level of significance.

^a^Data were expressed as number (%).

BMI, body mass index; Cr, creatinine; TG, triglycerides; TC, Total cholesterol; HDL, high-density lipoprotein; LDL, low-density lipoprotein.

The demographic and laboratory data of the groups are outlined in Table 1.

**Figure 1:**
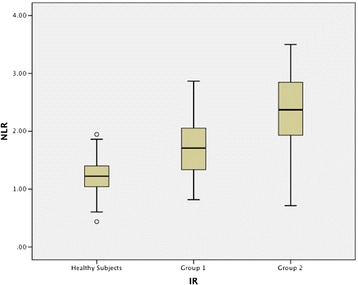
**Mean neutrophil-lymphocyte ratio (NLR) values of the groups.**

**Figure 2:**
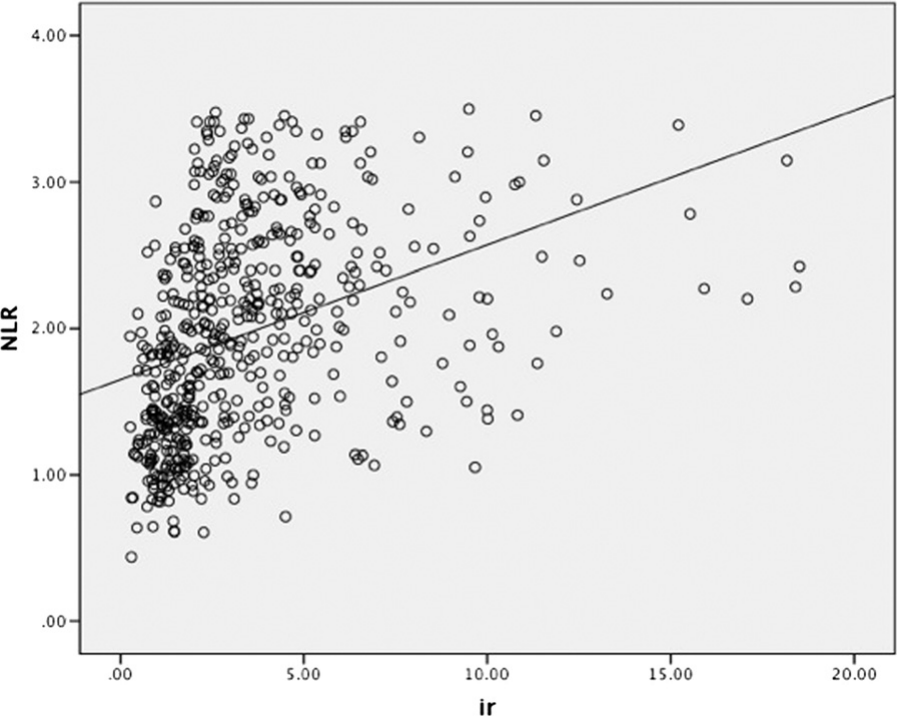
**The association between neutrophil-lymphocyte ratio (NLR) and insulin resistance (IR).**

The diabetic patients were divided into two groups according to their HOMA-IR score after the demographic and laboratory data evaluation. Group 1: HOMA-IR ≤ 2.0; group 2: HOMA-IR > 2.0 [19,20]. Group 2 was found in 310 of the 413 DM patients (75.1%). These groups had similar ages, and BMIs. NLR strongly correlated with neutrophil and lymphocyte values. Mean neutrophil values significantly increased and mean lymphocyte values decreased in group 2; hence, the NLR value was significantly higher in group 2 than in group 1 (Table 1, Figure 1). NLR showed significant positive correlation with HOMA IR (r = 0.285; P < 0.001) (Figure 2).

A logistic regression analysis was also carried out using the enter method to evaluate the risk factors for IR. The measurement of NLR, TG and HbA1c were dependent parameters, whereas age, gender, BMI, systolic blood pressure (SBP), diastolic blood pressure (DBP), total cholesterol (TC), HDL-C, LDL-C, and microalbuminuria were independent parameters. As shown in Table 2, the results showed that IR was independently related to NLR, TG and HbA1c.

**Table 2:** **Logistic regression analysis of factors independently associated with IR**

**Variable**	**P value**	**EXP(B)**	**95% CI**
NLR	<0.001	7.231	4.277–12.223
Gender	0.303	0.709	0.368–1.365
Age (years)	0.856	1.005	0.948–1.066
BMI (kg/m^2^)	0.257	1.045	0.969–1.126
SBP (mm Hg)	0.165	1.012	0.995–1.030
DBP (mm Hg)	0.858	1.003	0.970–1.038
TC (mmol/L)	0.691	1.078	0.744–1.562
TG (mmol/L)	0.030	1.338	1.028–1.741
HDL (mmol/L)	0.626	1.254	0.504–3.121
LDL (mmol/L)	0.667	1.111	0.689–1.792
HbA1c (%)	0.027	1.164	1.018–1.331
Microalbuminuria	0.571	0.997	0.988–1.007

CI, confidence interval; SBP, systolic blood pressure; DBP, diastolic blood pressure; HbA1c, glycated.

Hemoglobin.

P < 0.05 was accepted as the level of significance.

## Discussion

The present study had shown that the NLR values of the diabetic patients were significantly higher than those of the healthy control (P < 0.001), and the NLR values of the patients with a HOMA-IR value of > 2.0 are notably greater than those of the patients with a HOMA-IR value of ≤ 2.0 (P < 0.001).

Many epidemiological studies have determined that DM is associated with chronic inflammation [21], which may contribute to the acceleration of diabetic microangiopathy and the development of macroangiopathy [3,4]; IR is a characterized of T2DM, whereas the exact molecular action leading to IR is not yet understood, several studies have associated IR with inflammation [1,6] experimental studies have demonstrated a link between chronic inflammation and insulin resistance through mechanisms involving obesity [22] and atherosclerosis [23]. NLR has been recently defined as a novel potential inflammation marker in cancer and cardiovascular diseases [14,16]. NLR can easily be calculated using the neutrophil-lymphocyte ratio in peripheral blood count. Calculating NLR is simpler and cheaper than measuring other inflammatory cytokines, such as IL-6, IL-1β, and TNF-α [24].

In addition, NLR was found to be a significant risk factor for IR with DM through logistic regression analysis. The pathological activation of innate immunity leads to inflammation of the islet cells, resulting in a decrease in pancreatic beta-cell mass and impaired insulin secretion [25]. Patients with T2DM are in a state of low-degree chronic inflammation that induces hypersecretion of inflammatory factors, such as CRP, IL-6, TNF-α, and MCP-1, which results in a constantly elevated neutrophilic granulocyte count [26]. One mechanism by which increased levels of neutrophils could mediate IR may be through augmented inflammation. The increase in NLR appears to underlie the elevated levels of pro-inflammation, as evident from the persistent neutrophil activation and enhanced release of neutrophil proteases with T2DM [27]. Moreover, lymphocytes may be also associated with inflammation. Some studies have shown that IR may be related to the signal transduction mediated by T cells and that IR results in a decrease in T-cell count [28,29]. Figure 2 presents the scatter plot of Pearson correlation analysis.

However, some common physical conditions, such as dehydration and Prostate Specific Antigen of the blood specimen can affect the accuracy of the data. Furthermore, physical exercise and release of catecholamine (CA) can cause a drop in neutrophilic granulocyte and lymphocyte. NLR represents a combination of two markers where neutrophils represent the active nonspecific inflammatory mediator initiating the first line of defense, whereas lymphocytes represent the regulatory or protective component of inflammation [30]. NLR is superior to other leukocyte parameters (e.g., neutrophil, lymphocyte, and total leukocyte counts) because of its better stability compared with the other parameters that can be altered by various physiological, pathological, and physical factors [17,31]. Thus, as a simple clinical indicator of IR, NLR is more sensitive compared with the neutrophilic granulocyte count and CRP levels, which are widely used as markers of IR [32,33].

A logistic regression analysis of the following risk factors was conducted: NLR, TG and HbA1c. In our study, in conjunction with the rising of the level of HbAlc, the degree of IR increased significantly. HbA1c showed an association with early-phase insulin secretion assessed by insulinogenic index [34]. Heianza et al. [35] reported that elevated HbA1c levels of above 41 mmol/mol (>5.9%) were associated with a substantial reduction in insulin secretion and insulin sensitivity as well as an association with β-cell dysfunction in Japanese individuals without a history of treatment of diabetes. Increased accumulation of TG has been observed in human muscle tissue of obese and type 2 diabetic subjects, and associated with IR [36,37], which is in agreement with the present study. IR reduces the inhibition effect of lipolysis in adipose tissue, resulting in the increase of the free fatty acid (FFA) level in plasma. Plasma FFA levels usually increase in obesity [38]. Infusion of free fatty acids (FFA) has been shown to induce IR in skeletal muscles [39] in several studies. However, in the present study, NLR serves an important function in predicting the risk of IR. IR in diabetic patients is related to chronic inflammation, and NLR may be helpful in assessing the prognoses of these patients.

## Conclusion

We recommend that the NLR values of diabetic patients be calculated as NLR is a cheap, predictive, and prognostic marker for IR. High NLR values were independently related to IR.
